# Revealing the main factors and two-way interactions contributing to food discolouration caused by iron-catechol complexation

**DOI:** 10.1038/s41598-020-65171-1

**Published:** 2020-05-19

**Authors:** Judith Bijlsma, Wouter J. C. de Bruijn, Jos A. Hageman, Peter Goos, Krassimir P. Velikov, Jean-Paul Vincken

**Affiliations:** 1 Laboratory of Food Chemistry, Wageningen University & Research, Bornse Weilanden 9, 6700 AA Wageningen, The Netherlands; 2 Biometris, Applied Statistics, Wageningen University & Research, Droevendaalsesteeg 1, 6700 AA Wageningen, The Netherlands; 3 Faculty of Bioscience Engineering, KU Leuven, Kasteelpark Arenberg 30, 3001 Heverlee, Belgium; 4 Unilever Innovation Centre B.V. Bronland 14, 6708 WH Wageningen, The Netherlands; 5 Institute of Physics, University of Amsterdam, Science Park 904, 1098 XH Amsterdam, the Netherlands; 6 Soft Condensed Matter, Debye Institute for Nanomaterials Science, Utrecht University, Princetonplein 5, 3584 CC Utrecht, The Netherlands

**Keywords:** Chemistry, Coordination chemistry, Statistics

## Abstract

Fortification of food with iron is considered to be an effective approach to counter the global health problem caused by iron deficiency. However, reactivity of iron with the catechol moiety of food phenolics leads to discolouration and impairs bioavailability. In this study, we investigated the interplay between intrinsic and extrinsic factors on food discolouration caused by iron-catechol complexation. To this end, a three-level fractional factorial design was implemented. Absorbance spectra were analysed using statistical methods, including PCA, HCA, and ANOVA. Furthermore, a direct link between absorbance spectra and stoichiometry of the iron-catechol complexes was confirmed by ESI-Q-TOF-MS. All statistical methods confirm that the main effects affecting discolouration were type of iron salt, pH, and temperature. Additionally, several two-way interactions, such as type of iron salt × pH, pH × temperature, and type of iron salt × concentration significantly affected iron-catechol complexation. Our findings provide insight into iron-phenolic complexation-mediated discolouration, and facilitate the design of iron-fortified foods.

## Introduction

Iron deficiency is a global health problem, affecting one quarter of the world’s population^1^. Iron fortification of food is an effective solution to counter iron malnutrition^2^. However, due to reactivity of the ‘free’ iron ion, iron fortification of food is notoriously difficult. Especially in food that contains plant material, complexation between iron and phenolic compounds can compromise product colour and impair bioavailability of iron^3,4^. Savoury concentrates (*e.g*. bouillon cubes) are an example of a phenolic-containing food product that is a promising vehicle for iron fortification, as they are widely available, frequently consumed, and affordable^5,6^. Discolouration of iron-fortified savoury concentrates is currently limiting their successful introduction to the market, as colour is one of the critical sensory parameters of food^7^.

**Figure 1:**
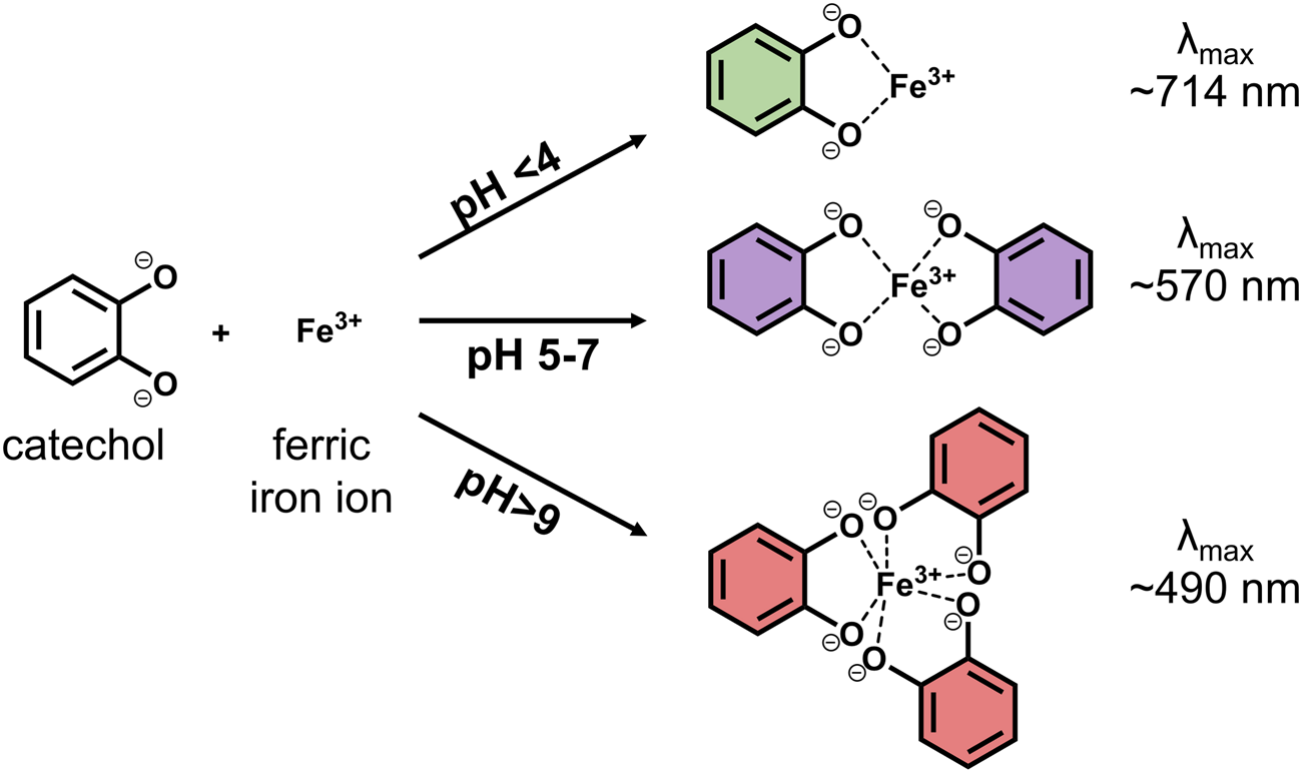
Coordination of ferric iron by catechol at different pH values and corresponding maximum absorbance (λ_max_) resulting from ligand-to-metal charge transfer (based on^7,12^).

To date, chemical interactions between phenolics and iron, combining redox processes and complex formation, have not been fully elucidated^8^. Oxidation of the 1,2-dihydroxybenzene (*i.e*. catechol) moiety of phenolics in presence of ferric iron (Fe^3+^) leads to the formation of quinones that polymerise to form brown-coloured compounds^4,8^. However, Fe^3+^-catalysed oxidation reactions of phenolic derivatives and catechol are very slow^9–11^. On the other hand, complexation reactions by the formation of a coordinate bond between Fe^3+^ and deprotonated catechol, lead to fast and intense discolouration. Discolouration upon iron-catechol complexation results from the intense ligand-to-metal charge transfer (LMCT) absorbance band, typically observed between 380–800 nm^12^. Deprotonation of the catechol moiety is required for iron binding, thus stoichiometry and colour of the iron-catechol complexes is pH dependent, as the complexing capacity of catechol increases at higher pH (Fig. 1)^8,10,12^. The pK_a_ for the first hydroxyl group of catechol is 9.3 and of the second hydroxyl group 13.0^13^. However, if iron is present, the deprotonated state is stabilised and thermodynamically more favourable, leading to an apparent pK_a_ of 5–8^14,15^.

Iron-mediated oxidation of catechol is a slow process^9^, therefore, the main mechanism responsible for discolouration of iron-fortified, phenolic-rich food products is expected to be iron-catechol complex formation. This study, therefore, focusses on discolouration resulting from fast complexation between iron and catechol.

Understanding the interplay between intrinsic and extrinsic factors on iron-catechol complexation is required to design iron-fortified products while preventing discolouration and ensuring bioavailability of iron. The extrinsic factors of interest for food systems are temperature and humidity^3,4^. Intrinsic factors expected to influence the iron-catechol complexation are: pH, the type of iron salt, iron concentration, ratio iron:catechol, ionic strength, and presence of taste enhancers^4,16^. The effect of several factors (*e.g*. temperature and ionic strength) on iron-catechol complexation is currently unknown or unclear due to contradictory reports in literature^3,4,17–19^. Additionally, two-way interactions between the aforementioned factors have not yet been investigated.

The goal of this study is to understand the effect of seven factors on iron-catechol complexation and discolouration to facilitate optimisation of conditions in iron-fortified food. To obtain systematic insight in the effect of the seven factors at three levels, and their combined effects, a model system of aqueous mixtures of catechol (1,2-dihydroxybenzene) was studied using a duplicated regular 3^8–3^ fractional factorial design (FrFD). It is hypothesised that the main effects contributing to iron-catechol complexation are type of iron salt, pH, and temperature. Additionally, two-way interactions between these factors, or with other factors are expected to contribute to iron-catechol complexation.

## Results and Discussion

### Exploratory analysis of factors affecting complexation

The combined effect of 7 factors at 3 levels (Table 1) on iron-catechol complexation was assessed by measuring the absorbance spectra for all 243 combinations obtained by the FrFD. In this study, the iron salts ferric phosphate (FePO_4_) and ferric pyrophosphate (Fe_4_(P_2_O_7_)_3_, hereafter FePP), which are commonly used for food fortification, were compared to the well-documented iron salt ferric chloride (FeCl_3_). For the other factors the levels were chosen based on relevance for food applications.

**Table 1:** Seven experimental factors and the corresponding three levels tested using the fractional factorial design.

Factors	Code	Level 0	Level 1	Level 2
Type of iron salt	**A**	FeCl_3_	FePO_4_	FePP
pH	**B**	3	5	8
Temperature (°C)	**C**	23	40	100
Ionic strength (mM NaCl)	**D**	0	100	1000
Concentration iron (mM)	**E**	1	5	10
Ratio [Fe]:[Cat]	**F**	1:1	1:2	1:3
MSG (mM)	**G**	0	125	250

**Figure 2:**
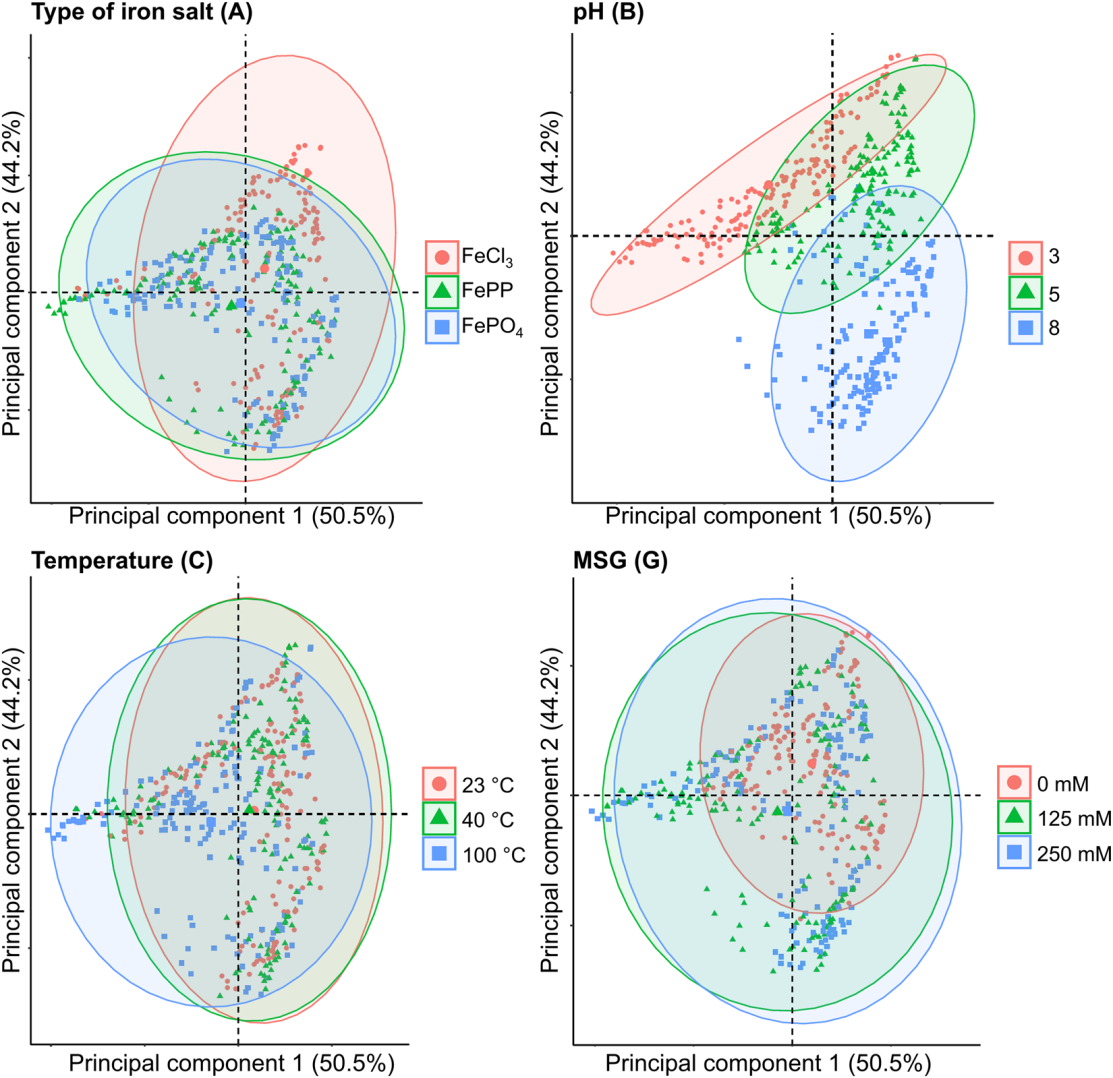
PCA biplots showing the principal component scores based on the normalised absorbance spectra, with colour indication for the different levels of the factors: type of iron salt (**A**), pH (**B**), temperature (**C**), and MSG (**G**).

To create a concise overview of the different absorbance spectra, two exploratory statistical analyses were performed: principal component analysis (PCA) and hierarchical cluster analysis (HCA). Analysis by PCA provides an unbiased overview of the absorbance spectra, and simplifies the different factors and levels by reducing the data dimensionality. The first two principal components accounted for 95% of the total variance. In the plot of PC1 vs. PC2, the three levels of each factor were defined and evaluated. The PCA plots indicated that the factors type of iron salt (**A**), pH (**B**), temperature (**C**), and presence of MSG (**G**) were the main effects defining the location of the sample in the PC1-PC2 plane (Fig. 2). However, the three levels could not be discriminated entirely for these factors. For the factors ionic strength (**D**), iron concentration (**E**), and ratio iron:catechol (**F**), no clear differences between the levels were observed (Supplementary I, Fig. SI-1).

**Figure 3:**
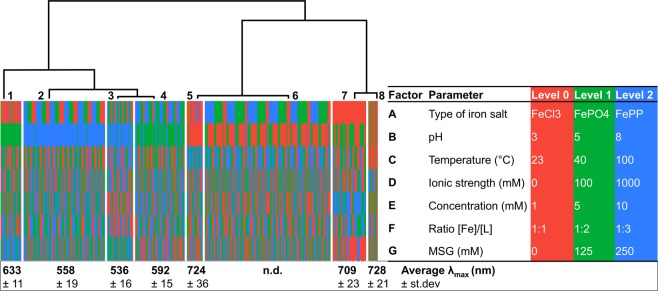
Dendrogram of samples hierarchically clustered based on the normalised absorbance spectra. The heatmap indicates the different levels of factors for each cluster as shown in the table. The average maximum absorbance wavelength (λ_max_) for each cluster is indicated below the heatmap. n.d. indicates that no LMCT band was detected.

For better interpretation of the PCA data, and to visualise which factors were most influential, HCA was used to cluster the normalised absorbance spectra. Figure 3 represents the dendrogram obtained from HCA, which depicts the relationships between absorbance spectra and experimental factors. These results indicated that the samples could be objectively grouped into 8 distinct clusters with similar absorbance spectra (Supplementary I, Fig. SI-2). Colours in the heatmap below the dendrogram, correspond with the different factors and levels as indicated in Fig. 3. Based on HCA, differences among the clusters were mainly the result of varying levels in the factors pH and type of iron salt. After clustering the average λ_max_ value per cluster was calculated to obtain additional information regarding colour and complexation for each specific cluster. Typically, the LMCT absorbance band for iron-catechol complexes is from 380–800 nm^12^. Based on the λ_max_ values of the clusters, 1:1 complexes (~714 nm) were expected in cluster **5**, **7** and **8**, 1:2 complexes (~570 nm) in cluster **1**, **2** and **4**, and a mixture of 1:2 (~570 nm) and 1:3 (~490 nm) in cluster **3**^8,10^. For the samples in cluster **6** no LMCT absorbance was observed (Supplementary I, Fig. SI-2). The heatmap in Fig. 3 shows that cluster **6** mainly contains samples at pH 3. This observation is explained by the reduced complexation affinity of catechol at pH < pK_a_. In addition to this, some samples in cluster **6** showed presence of white sediment, most likely due to the poor water solubility of the iron salts under the acidic conditions present in this cluster.

### Stoichiometry in the clusters confirmed by ESI-Q-TOF-MS

**Figure 4:**
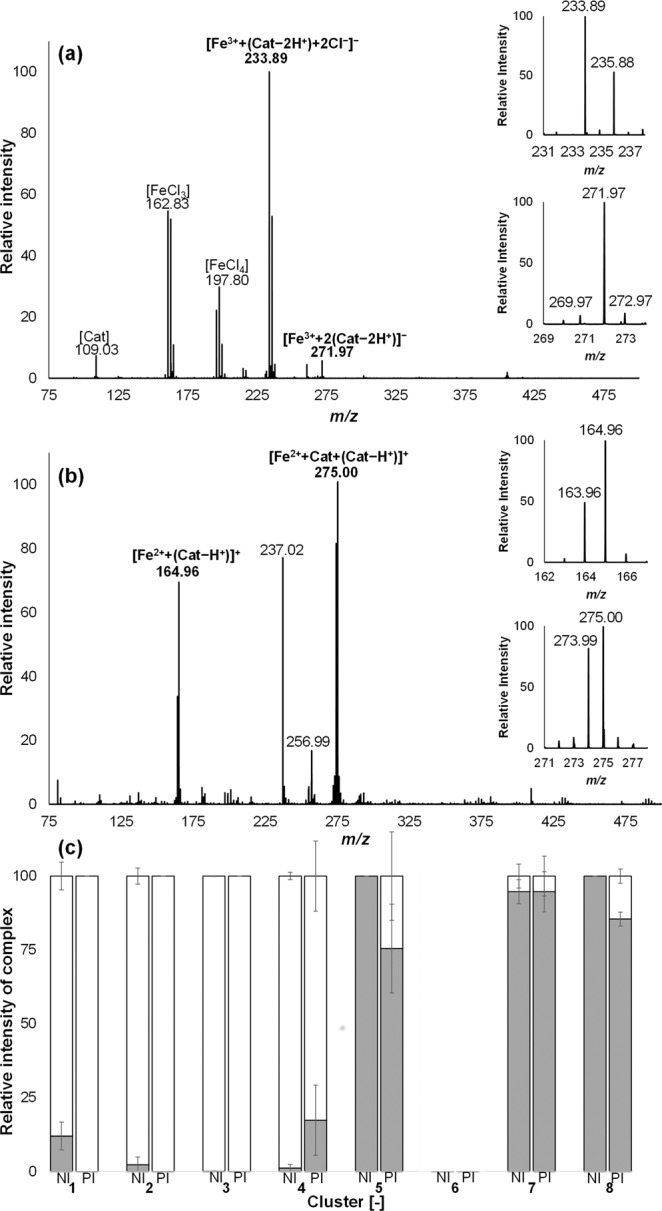
Electrospray ionisation mass spectra of a solution of ferric chloride (FeCl_3_) and catechol, recorded in (**a**) negative (NI) and (**b**) positive (PI) ionisation mode. The insets represent the isotopic patterns of the monocatecholate and dicatecholate complexes. (**c**) Relative intensities of monocatecholate (grey) and dicatecholate (white) iron complexes in each of the hierarchical clusters (Fig. 3) as measured in negative (NI) and positive (PI) ionisation mode (n = 3, error bar is relative standard deviation). For cluster 6 only traces of complex/or no complex at all was observed in the mass spectra.

Analysis by ESI-Q-TOF-MS was used to investigate whether the maximum absorbance of each hierarchical cluster could indeed be linked to the stoichiometry of the complexes present in the samples from that cluster. To this end, three samples from each cluster were measured in negative (NI) and positive ionisation (PI) mode mass spectrometry. It was previously shown that due to the soft character of ESI, iron-phenolic complexes remain intact^16,20^. Complexes of catechol with iron were detected at *m/z* 233.89, and 271.98 in NI (Fig. 4A) and *m/z* 163.96, 164.96, 273.99, and 275.00 in PI mode (Fig. 4B). These signals were absent in the FeCl_3_, FePP, FePO_4_, catechol, and MSG blanks. The iron-catechol complexes were tentatively identified based on the *m/z* value and isotope pattern (Table 2). Monocatecholate complexes were annotated in NI as chloride adduct at a *m/z* of 233.89 [Fe^3+^ + (catechol − 2 H^+^) + 2Cl^−^]^−^ and in PI at *m/z* 163.96 [Fe^3+^ + (catechol − 2 H^+^)]^+^ or *m/z* 164.96 [Fe^2+^ + (catechol−H^+^)]. Dicatecholate complexes were annotated in NI at *m/z* 271.98 [Fe^3+^ + 2(catechol − 2 H^+^)]^−^ and in PI at *m/z* 273.99 [Fe^3+^ + 2(catechol − H^+^)]^+^ or at *m/z* 275.00 [Fe^2+^ + catechol + (catechol − H^+^)]^+^. These annotations are in agreement with previously reported complexes of Fe^3+^ with phenolic ligands such as catechol^21^ or flavonoids^22,23^, using ESI-MS^21–23^. It is suggested that the complexes with Fe^2+^, as observed in PI, are a result of in-source charge reduction. In-source charge reduction reactions during ESI-MS in positive ion mode were reported previously for metal complexes^24,25^. Reduction of metal complexes in PI is a result of charge-transfer reactions between the iron complex and solvent molecules in the gas phase. Charge reduction was not observed for the iron-catechol complexes in NI.

**Table 2:** Tentative structures of iron-catechol complexes with corresponding experimental and theoretical mass-to-charge ratios (*m/z*) and isotope abundance.

Tentative Structures	Isotopes	*m/z*		Isotope abundance (%)	
		Exp.	Theoretical	Exp.	Theoretical
[Fe^3+^+(Cat − 2 H^+^) + 2Cl^−^]^−^	^12^C_6_H_4_O_2_^35^Cl_2_^54^Fe	231.89	231.90	2.3	6.4
	^12^C_6_H_4_O_2_^35^Cl_2_^56^Fe	233.89	233.89	100	100
	^12^C_5_^13^CH_4_O_2_^35^Cl_2_^56^Fe and ^12^C_6_H_4_O_2_^35^Cl_2_^57^Fe	234.89	234.90	4	6.5
	^12^C_6_H_4_O_2_^35^Cl^37^Cl^56^Fe	235.88	235.89	51.9	63.9
	^12^C_5_^13^CH_4_O_2_^35^Cl^37^Cl^56^Fe	236.89	236.89	2.1	4.1
	^12^C_6_H_4_O_2_^37^Cl_2_^56^Fe	237.88	237.89	4.5	10.2
[Fe^3+^+2(Cat − 2 H^+^)]^−^	^12^C_12_H_8_O_4_^54^Fe	269.97	269.98	7.3	6.4
	^12^C_12_H_8_O_4_^56^Fe	271.97	271.97	100	100
	^12^C_11_^13^CH_8_O_4_^56^Fe and ^12^C_12_H_8_O_4_^57^Fe	272.97	272.98	13.4	13
[Fe^2+^+(Cat − H^+^)]^+^	^12^C_6_H_5_O_2_^54^Fe	162.97	162.97	3.2	6.4
	^12^C_6_H_5_O_2_^56^Fe	164.96	164.96	100	100
	^12^C_5_^13^CH_5_O_2_^56^Fe and ^12^C_6_H_5_O_2_^57^Fe	165.97	165.96	4.2	6.5
[Fe^3+^+(Cat − 2H^+^)]^+^	^12^C_6_H_4_O_2_^54^Fe	161.96	161.96	3	6.4
	^12^C_6_H_4_O_2_^56^Fe	163.96	163.96	100	100
	^12^C_5_^13^CH_4_O_2_^56^Fe and ^12^C_6_H_4_O_2_^57^Fe	164.96	164.96	8.8	6.5
[Fe^2+^+Cat + (Cat − H^+^)]^+^	^12^C_12_H_11_O_4_^54^Fe	272.99	273.00	8.1	6.4
	^12^C_12_H_11_O_4_^56^Fe	275.00	275.00	100	100
	^12^C_11_^13^CH_11_O_4_^56^Fe and ^12^C_12_H_11_O_4_^57^Fe	276.00	276.00	10	13
[Fe^3+^+(2Cat − H^+^)]^+^	^12^C_12_H_10_O_4_^54^Fe	271.98	272.00	7.3	6.4
	^12^C_12_H_10_O_4_^56^Fe	273.99	273.99	100	100
	^12^C_11_^13^CH_10_O_4_^56^Fe and ^12^C_12_H_10_O_4_^57^Fe	275.00	275.00	15.3	13

Based on the results of ESI-Q-TOF-MS analysis, an overview was made of the distribution of stoichiometry of the complexes in each cluster (Fig. 4C). Samples in cluster **5**, **7**, and **8** showed mainly 1:1 stoichiometry, these outcomes are in agreement with the average λ_max_ value of those clusters between 709 and 728 nm (Fig. 1). The samples in cluster **1**, **2**, and **4** were mainly 1:2 with minor amounts of 1:1, which matches with their average λ_max_ (between 558 and 633 nm). Samples in cluster **3** showed only 1:2 stoichiometry, consistent with the average λ_max_ of 536 nm. Generally, a mixture of 1:2 and 1:3 metal to ligand species are present in the pH range of 7–9^8^. However, no tricatecholate complexes were detected in any of our samples. Based on the average λ_max_ values of our clusters a mixture of 1:2 and 1:3 complexes was only expected in cluster **3** (average λ_max_ 536 ± 16). For the samples of cluster **6**, only trace amounts of the 1:1 complex could be observed, which is in agreement with the absence of an LMCT band in the absorbance spectra (Supplementary, Fig. SI-2).

### Factors affecting iron-catechol complexation

**Figure 5:**
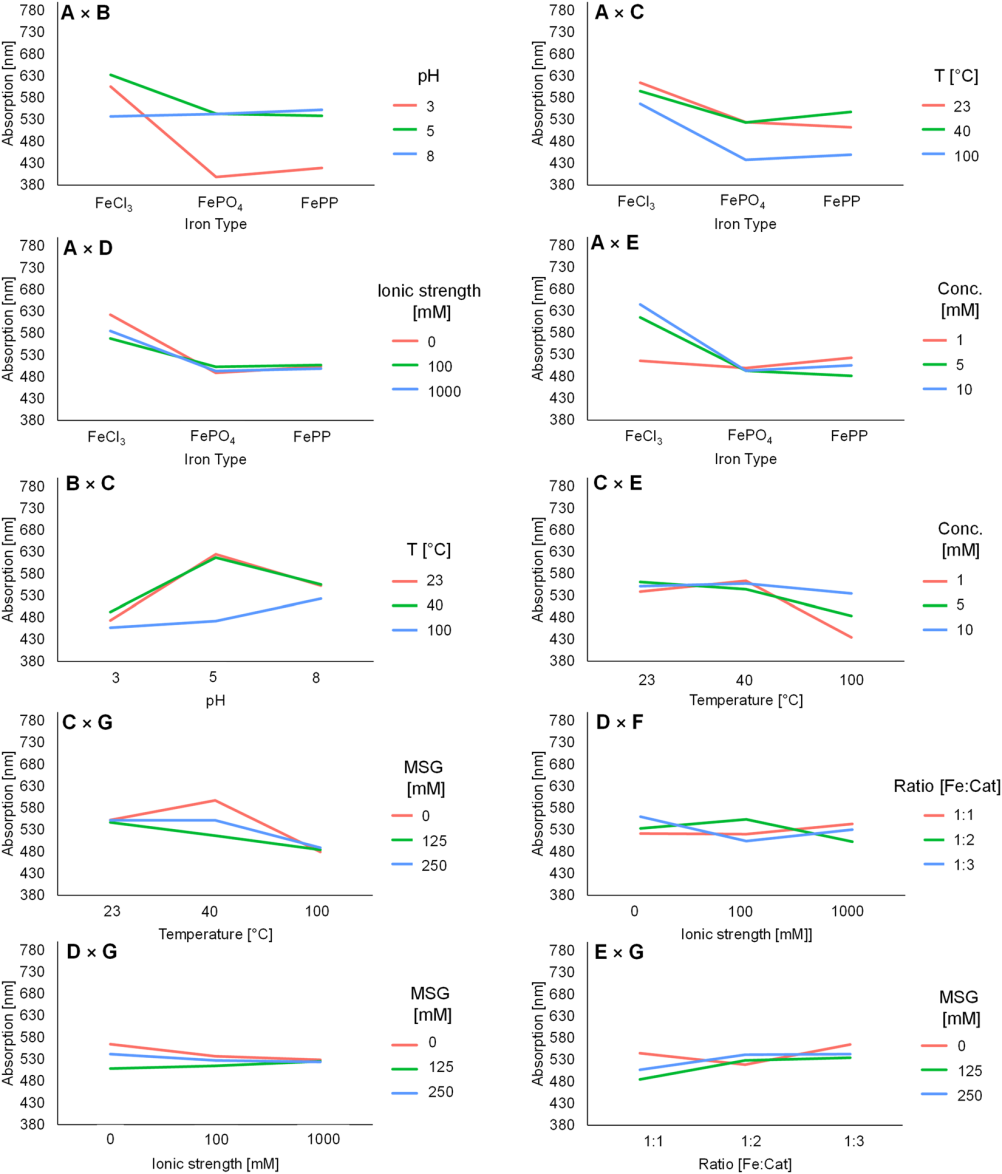
Interaction plots for the significant two-way interactions (*p* < 0.05) given by ANOVA (n = 486), average maximum absorption wavelength is indicated on the y-axis. Letters indicate the factors corresponding with Table 1.

The results from PCA and HCA provide an unbiased overview of the main factors that affect the full absorbance spectra. To gain more in-depth insight concerning the influence of the 7 factors’ main effects and 21 two-way interactions on iron-catechol complexation, analysis of variance (ANOVA) was performed (Table 3). ANOVA requires a quantitative dependent variable, to this end, the λ_max_ value of each independent sample was used. Absorbance intensity was not taken into account. The ANOVA results (Table 3) confirmed that several two-way interactions significantly (*p* < 0.05) contributed to changes in λ_max_, and therefore affected discolouration. Significant two-way interactions were observed for **AB**, **AC**, **AD**, **AE**, **BC**, **CE**, **CG**, **DF**, **DG**, and **EG**. Together the two-way interactions explained 51% of the variance observed in λ_max_ value. Interaction plots for these significant two-way interactions are shown in Fig. 5.

**Table 3:** Analysis of variance of the 7 main effects and 21 interaction effects affecting iron-catechol complexation, significant factors and corresponding *p-*values are indicated in bold (R^2^ = 0.76 and R^2^_adj_ = 0.70).

Factor	Sum of Squares	Mean Square	F Value	*p-*value	Contribution (%)
**A** (type of iron salt)	937383	468691	115	**6.68 × 10**^**−40**^	19
**B** (pH)	811126	405563	99	**1.42 × 10**^**−35**^	16
**C** (Temperature)	509469	254735	62	**3.11 × 10**^**−24**^	10
D (Ionic strength)	16304	8152	2	0.14	0
**E** (Concentration iron)	100814	50407	12	**6.27 × 10**^**−6**^	2
F (Ratio [Fe]:[Cat])	869	434	0	0.90	0
**G** (MSG)	59483	29742	7	**7.78 × 10**^**−4**^	1
**A × B**	780182	195045	48	**1.15 × 10**^**−32**^	16
**A × C**	90012	22503	6	**2.50 × 10**^**−4**^	2
**A × D**	73998	18500	5	**1.36 × 10**^**−3**^	1
**A × E**	442251	110563	27	**7.57 × 10**^**−20**^	9
A × F	2235	559	0	0.97	0
A × G	9240	2310	1	0.69	0
**B × C**	357090	89272	22	**2.66 × 10**^**−16**^	7
B × D	32866	8216	2	0.09	1
B × E	7930	1982	0	0.75	0
B × F	11954	2989	1	0.57	0
B × G	26167	6542	2	0.17	1
C × D	5424	1356	0	0.86	0
**C × E**	193111	48278	12	**4.41 × 10**^**−9**^	4
C × F	33035	8259	2	0.09	1
**C × G**	121354	30338	7	**8.83 × 10**^**−6**^	2
D × E	8845	2211	1	0.70	0
**D × F**	157640	39410	10	**1.86 × 10**^**−7**^	3
**D × G**	40674	10169	2	**0.04**	1
E × F	10606	2651	1	0.63	0
**E × G**	81992	20498	5	**5.86 × 10**^**−4**^	2
F × G	16030	4008	1	0.42	0

The two-way interactions that explained more than 5% of the variance (**AB**, **AE**, and **BC**) are further discussed in the sections below. For the other significant two-way interactions, the mechanisms underlying their effect have not yet been elucidated. To the best of our knowledge, previous studies on the effect of various factors on discolouration caused by iron-phenolic complexation did not take two-way interactions into account^3,4^.

The obtained ANOVA model can be used to predict the λ_max_ value of unknown combinations of factors (R^2^_adj_ = 0.70). The parameter estimates of the prediction model are presented in Supplementary II (Table SII-1).

#### Effect of iron salt, pH, and temperature on discolouration

Significant interaction effects were observed between the type of iron salt (**A**) and pH (**B**). Generally, it was observed that mixtures with FeCl_3_ resulted in discolouration at all pH values, although the λ_max_ varied depending on pH. At pH 8 a significant decrease in λ_max_ was observed compared to pH 3 or 5 (Tukey *p*-values of 1.18 × 10^−4^ and 1.03 × 10^−7^, respectively). This is in line with the enhanced complexing capacity at pH > pK_a_. For FePP and FePO_4_, a significant increase in discolouration was observed at pH > 3 (Fig. 5; Supplementary II, Table SII-2). To verify this conclusion, confirmatory experiments were performed with only the iron salt and pH as variable factor. In these experiments, iron-catechol mediated discolouration was observed for FeCl_3_ at all pH values. For the FePP and FePO_4_ samples at pH 3, no colour was observed and white sediment (*i.e*. precipitate from undissolved iron salt) was present (Supplementary I, Fig. SI-3). We believe that the interplay between the type of iron salt and pH (**AB**) could be explained by the enhanced dissolution of the iron ion from FePP and FePO_4_ upon an increase in pH^26,27^, whereas the iron ion from FeCl_3_ is readily soluble at pH 3. Enhanced dissolution of the iron ion from FePP and FePO_4_ upon increasing pH may either result from weaker interactions of phosphate and pyrophosphate ions with Fe^3+^ at elevated pH and/or from the formation of soluble complexes with deprotonated catechol^28,29^. Tukey’s *post hoc* comparison (Supplementary II, Table SII-2) also showed that, at pH 3 and 5, FeCl_3_ significantly differed from FePP and FePO_4_ (Tukey*, p* < 0.05). On the other hand, no significant difference between the two poorly soluble iron salts FePP and FePO_4_ was observed at any pH. These outcomes indicate the importance of the type of iron salt used for food fortification in relation to the pH of the food product.

The interaction pH × temperature (**BC**) also significantly affected complexation. This is contrary to a previous report in which the temperature effect did not depend on pH for a combination of Fe^2+^ and flavanols^3^. Habeych and co-authors state that colour development and formation of a dark precipitate was observed after heat treatment at 95 °C, independently of pH. To further investigate the combined effect of pH and temperature, we performed additional experiments with pH and temperature as the only two independent variables, which showed a hypochromic shift in absorbance at pH 3 and 5 upon an increase of temperature and a hypsochromic shift at pH 8 (Supplementary I, Fig. SI-4). *Post hoc* comparisons show no significant difference between the temperatures at pH 3 and 8. At pH 5 no significant difference was observed between 23 °C and 40 °C, but the results at 100 °C were significantly different (Supplementary II, Table SII-3), in agreement with PCA results (Fig. 2). In some of the samples treated at 100 °C, a dark precipitate was observed (Supplementary I, Fig. SI-5). Likely, this precipitate results from iron-catalysed oxidation reactions that occur in parallel to, or as a continuation on the complexation reaction. Besides temperature, oxidation of phenolics is influenced by pH^30^, possibly explaining the interaction between pH and temperature observed in this study. In a previous study, Fe^3+^-catalysed oxidation of phenolic derivatives at room temperature was shown to be slow^9^. Faster oxidation at elevated temperatures is expected, as oxidation is a first-order reaction following a direct relation with temperature, as dictated by the Arrhenius equation. Further studies are necessary to gain more in-depth mechanistic insight in the effect of the two-way interaction of pH and temperature on iron-catechol complexation and the possible oxidation in iron-fortified food under various conditions. The outcomes of this study implicate that iron-catechol complexation may not be affected in regions with warmer climates (~40 °C), but is affected upon boiling (100 °C).

#### Effect of iron and catechol concentration on absorbance

The interaction effect between concentration and type of iron salt (**AE**) significantly contributed to discolouration. Colour development (intensity) was previously shown to be proportional to the iron concentration^4^. However, the λ_max_ was not expected to vary with the iron salt concentration. Tukey’s test showed significant differences for FeCl_3_ at the lowest concentration tested (Supplementary II, Table SII-4). The mechanism behind this effect is unclear.

#### Addition of MSG affects iron-catechol complexation

Interestingly, monosodium glutamate (MSG) showed significant interaction effects with temperature, ionic strength and concentration. PCA also showed that addition of MSG affected the absorption spectra (Fig. 2). Binding of glutamate to Fe^3+^ was reported previously^31,32^. However, the effect was expected to be minimal as Fe^3+^ has a lower overall binding stability constant for glutamate (Log β = 5.5) compared to the catechol groups of phenolics (Log β = 43.8)^31–33^. Competitive or ternary complexation between iron, catechol, and MSG was further investigated using ESI-Q-TOF-MS. In these analyses, a peak was observed at *m/z* 308.98 in negative ionisation mode, which was only present in the samples that contained the combination of iron, catechol, and MSG. Based on the *m/z*, isotope pattern, and fragmentation spectra, this peak was tentatively identified as a ternary complex of catechol and iron with glutamate (Supplementary I, Table SI-1**)**. Glutamate most likely interacts with iron via the amino nitrogen and the carboxylate oxygen^34^. The difference in absorbance (λ_max_) upon formation of the ternary complex may be due to the weaker interaction of glutamate with Fe^3+^ compared to catechol. Because of this, the λ_max_ value of the ternary complex is expected to be between that of a 1:1 and 1:2 complex^12^. In line with this hypothesis, the λ_max_ of cluster **1**, which mainly contains the samples with high concentrations of MSG (Fig. 3) shows a hypsochromic shift compared to 1:1 and a bathochromic shift compared to 1:2 complexes. Further research regarding the apparent stability of the ternary complex is needed to confirm this hypothesis.

### Mechanisms of discolouration by iron-catechol reactions

The focus of this study was on the formation of water-soluble iron-catechol complexes, under a wide variety of conditions, and the influence of this phenomenon on discolouration. Besides iron-catechol complexation, discolouration could also originate from iron-catalysed oxidation and polymerisation of catechol^8^ or from the formation of the strongly light absorbing iron oxides and hydroxides upon Fe^3+^ hydrolysis^35^. The latter is expected to be limited in the tested samples, as fast complexation of catechol to Fe^3+^ stabilises against formation of Fe^3+^ hydrolysis products^36,37^. Moreover, neither ferric oxides and hydroxides, nor polymeric catechol oxidation products, were detected in the UV-Vis and MS spectra. Despite this, after incubation for 1 hour, black precipitate formation was observed in some samples, especially at elevated temperatures (Supplementary I, Fig. SI-5). Precipitate formation is suggested to result from the formation of insoluble products upon iron-catalysed catechol oxidation. In absence of iron, catechol can undergo autoxidation reactions in aqueous solution around neutral pH^13^. The autoxidation products formed in the absence of iron are soluble and brown-coloured, whereas a clear black colour was observed of the precipitate in presence of iron (Supplementary I, Fig. SI-5), indicating that different products were formed upon iron-catalysed oxidation. The observation that catechol is already oxidised after 1 hour in presence of iron is contrary to our initial expectations based on previous reports, which describe slow Fe^3+^-catalysed oxidation rates of phenolic derivatives^9^. The faster catechol oxidation rate observed in this study may result from elevated temperatures or different pH. Alternatively, the precipitate could be a result of the formation of iron-catechol networks^38^. These networks between Fe^3+^ and phenolic derivatives can be formed thermodynamically at elevated temperature or due to kinetic assembly at low temperature^39^. The findings of this study indicate that precipitation might occur, depending on intrinsic and extrinsic factors, which warrants further research on the parallel occurrence of iron-phenolic complexation and oxidation.

### Implications for iron fortification of foods

As many common food phenolics also contain a catechol moiety, the findings of this study can be applied to the design of iron-fortified food formulations. The outcomes in this study indicate that the combined effect of factors on iron-phenolic complexation should not be neglected in the development of iron-fortified food. Food production, storage, and preparation are dynamic processes during which intrinsic (*e.g*. pH) and extrinsic factors (*e.g*. temperature) continuously change, resulting in discolouration depending on product properties, as shown by the findings of this study. Implementation of the parameter estimates from the ANOVA model that was generated by this study (Supplementary II, Table SII-1) can be useful to predict the λ_max_ value of previously untested combinations of factors in the design of novel iron-fortified foods. Nevertheless, it should be considered that this prediction model is based on a model system and should be validated for real food formulations.

Our results indicate that usage of a combination of poorly soluble iron salts and low pH is a promising approach to limit discolouration due to iron-phenolic complexation. However, the effect of low pH on the organoleptic properties of food and bioavailability of iron should also be considered. Even though the iron salts FePP and FePO_4_ show poor solubility and low reactivity at acidic pH, the bioavailability of the iron ion is not necessarily affected by low pH. Most of the iron is absorbed in the duodenum (pH 6-6.5) and upper jejenum (pH 7–9)^40^. In these pH ranges, solubility of the iron ion from FePP and FePO_4_ is increased compared to low pH, enhancing the iron bioavailability at the location of iron absorption^27,41^. In this study, savoury concentrates are mentioned as example of iron-fortified food. Besides, the findings provided can also be implemented in the design of other iron-fortified phenolic containing food products.

## Conclusions

In this study, we successfully implemented a fractional factorial design to gain systematic insight in the combined effect of 7 experimental factors at 3 levels on iron-catechol complexation reactions and resulting discolouration in an iron-fortified food model system. HCA was used to identify 8 hierarchical clusters, each with different absorbance spectra resulting from varying stoichiometries of iron-catechol complexes, as confirmed by ESI-Q-TOF-MS. All three statistical methods revealed that the most important factors in iron-catechol complexation were type of iron salt, pH, and temperature. Additionally, the interactions of several factors, such as type of iron salt × pH, pH × temperature, and type of iron salt × concentration significantly affect iron-catechol complexation and are of practical importance. These interaction effects should not be neglected in the development of iron-fortified food. To minimise discolouration in food due to iron-phenolic complexation, application of the iron salts FePP or FePO_4_ in combination with low pH seems most promising.

## Materials and Methods

### Materials

Ferric pyrophosphate (FePP) was obtained from Dr. Paul Lohmann GmbH KG (Emmerthal, Germany). All other chemicals used were purchased from Sigma Aldrich (St. Louis, MO, USA). Water was prepared using a Milli-Q water purification system (Merck Millipore, Billerica, MA, USA).

### Experimental design

The combined effect of 7 different factors at 3 levels was investigated in this study. To limit the required number of experiments, while maximising the information obtained, a duplicated regular 3^8–3^ fractional factorial design (FrFD) with resolution V was implemented. This FrFD allowed us to estimate the main independent effects and the two-factor interactions that affect discolouration by iron-catechol complexation. The orthogonal array L_243_ (3^8–3^) was designed according to design 8–3.1^42^. The first factor in the design was the well plate in which the sample was tested. The seven other factors studied were: (**A**) type of iron salt, (**B**) pH, (**C**) temperature, (**D**) ionic strength, (**E**) concentration of iron, (**F**) iron:catechol ratio, and (**G**) presence of MSG. The factor humidity was not taken into account as aqueous mixtures were tested. Each factor was studied at 3 levels; the factors with their respective levels are shown in Table 1. A total of 243 test combinations were investigated. Each of these were duplicated independently.

### Preparation of iron-catechol mixtures

Mixtures of FeCl_3_, FePO_4_, and FePP (final concentration iron ion 10 mM) were prepared in Eppendorf tubes and combined with catechol, sodium chloride, and MSG solutions according to the orthogonal array. Concentrated HCl and NaOH were used to adjust the pH as buffer usage is known to interfere with the complexation reaction^43,44^. Samples were incubated at 23, 40, or 100 °C for 1 hour under constant shaking at 1000 rpm.

### Colour assessment

After centrifugation for 5 min at 15,000 × *g*, colour of the samples was optically assessed and supernatant (200 µL) was transferred to a flat bottom 96 well-plate. Samples were diluted with water if needed. Dilution of sample was found to have a negligible effect on the absorbance spectra, except for the expected decrease in intensity (data not shown). Visible light spectra were recorded in the range from 380 to 800 nm in a SpectraMax M2e (Molecular Devices, Sunnyvale, CA, USA), at room temperature.

### Statistical data analysis

To analyse the acquired absorbance spectra, multivariate statistical analyses were performed. Prior to analysis, sum of squares normalisation was performed on the raw absorbance spectra to normalise signal intensity. Principal component analysis (PCA) was carried out to reduce the data dimensionality and to visualise relations between the experimental factors and normalised absorbance spectra. In addition, hierarchical cluster analysis (HCA) using Euclidean distance and Ward’s linkage method^45^ was applied to depict similarities between the normalised absorbance spectra. HCA is an unsupervised clustering method where individual samples are combined into clusters based on similarity of their absorbance spectra. Normalisation, PCA, and HCA were performed by using the R statistical software package (R Core Team, 2013). The average maximum absorbance wavelength (λ_max_) of each hierarchical cluster was determined in SPSS, outliers (*z*-score> 3.0 or *z*-score < −3.0) were removed from the dataset.

In addition to the exploratory statistical methods described above, the significance of the individual factors and interactions were investigated quantitatively using analysis of variance (ANOVA). PCA and HCA were performed on the complete normalised absorbance spectra. ANOVA requires a quantitative dependent variable. Therefore, the λ_max_ value of the normalised spectra (n = 486) was used instead of the complete spectra. For statistical purposes, a λ_max_ of 380 nm (iron) or 410 nm (in presence of MSG) was used for the samples without a clear LMCT band. Tukey’s *post hoc* comparisons (significant at *p* < 0.05) were carried out to create better insight in the effect of the different levels for the significant factors. ANOVA analysis was performed using IBM SPSS Statistic v.23 software (SPSS Inc., Chicago, IL, USA).

### Evaluation of iron-catechol complexes by electrospray ionisation time of flight mass spectrometry (ESI-Q-TOF-MS)

Three samples per hierarchical cluster were randomly selected to be evaluated by ESI-Q-TOF-MS to further investigate the molecular structure of the iron-catechol complexes. Sample was introduced by direct infusion (2 mL h^−1^) on a Synapt G2-Si high definition time of flight mass spectrometer equipped with a z-spray electrospray ionisation (ESI) source (Waters, Milford, MA, USA). The instrument was externally calibrated with sodium iodide and operated in normal resolution mode. The capillary voltage was set to 3.0 kV and 1.8 kV in positive (PI) and negative ionisation mode (NI), respectively. The sample cone was operated at 30 V and 40 V for PI and NI, respectively, with the source temperature set at 150 °C. MS and MS^2^ spectra were acquired between *m/z* 25–800 for 2 min at a 0.3 s scan time. The trap collision energy was 6 V in single MS mode and 22 V in MS^2^ mode. Data acquisition and analysis were carried out by MassLynx v.4.1 (Waters, Milford, MA, USA).

## Supplementary information

Supplementary information.

Supplementary information2.

## Data Availability

The datasets generated during and/or analysed during the current study are available from the corresponding author on reasonable request.
