# Supplementary material for: Revealing the main factors and two-way interactions contributing to food discolouration caused by iron-catechol complexation
Source: Sci Rep. 2020 May 19;10:8288. doi: 10.1038/s41598-020-65171-1 (PMC7237488; doi:10.1038/s41598-020-65171-1)
Supplement: Supplementary file 1 — Supplementary information. [file 41598_2020_65171_MOESM1_ESM.docx]

Supplementary information I

**Revealing the main factors and two-way interactions contributing to food discolouration caused by iron-catechol complexation**

Judith BIJLSMA^1^, Wouter J.C. DE BRUIJN^1^, Jos A. HAGEMAN^2^, Peter GOOS^3^, Krassimir P. VELIKOV^4,5,6^, Jean-Paul VINCKEN^1,*^

^1^ Laboratory of Food Chemistry, Wageningen University & Research, Bornse Weilanden 9, P.O. Box 17, 6700 AA, Wageningen, The Netherlands

^2^ Biometris, Applied Statistics, Wageningen University & Research, Droevendaalsesteeg 1, P.O. Box 16, 6700 AA, Wageningen, The Netherlands

^3^ Faculty of Bioscience Engineering, KU Leuven, Kasteelpark Arenberg 30, Box 2456, 3001 Heverlee, Belgium.

^4^ Unilever Innovation Centre B.V. Bronland 14, 6708 WH Wageningen, The Netherlands

^5^ Institute of Physics, University of Amsterdam, Science Park 904, 1098 XH Amsterdam, the Netherlands

^6^ Soft Condensed Matter, Debye Institute for Nanomaterials Science, Utrecht University, Princetonplein 5, 3584 CC Utrecht, The Netherlands

^*^Corresponding author: [jean-paul.vincken@wur.nl](mailto:jean-paul.vincken@wur.nl)

Figure SI-1. Biplots showing the principal component scores based on the absorbance spectra, with colour indicating different levels of the corresponding factors: ionic strength (D), concentration of iron (E), and ratio [Fe]:[Cat] (F).

Figure SI-2. Overview of the clustered absorbance spectra after normalisation and HCA, the numbers 1-8 correspond with the cluster numbers of the dendrogram obtained by HCA (Figure 3).

**Figure SI-3.** Photographs of Eppendorf showing colour formation of catechol in combination with the different iron salts at pH ranging from 3-10 after 1 hour incubation at 23 °C.

Figure SI-4. Absorbance spectra of FeCl_3_-Catechol mixtures at (a) pH 3, (b) pH 5, and (c) pH 8 after incubation at 23 °C (solid line), 40 °C (dashed line), and 100 ° C (dotted line) for 1 hour.

Figure SI-5. Photographs of Eppendorf tubes showing colour formation and precipitation of iron-catechol mixtures (10 mM) at pH 3, 5, and 8 after 1 hour incubation at 23 °C or 100 °C. A catechol solution without iron (pH 8) after 1 hour incubation is shown as a reference.

Table SI-1. Tentative structure of the iron-catechol-glutamate complex with experimental and theoretical mass-to-charge ratios (*m/z*) and isotope abundance.

|  |  | *m/z* | |  | Isotope abundance (%) | | |
| --- | --- | --- | --- | --- | --- | --- | --- |
| **Tentative Structure** | **Isotopes** | **Exp.** | **Theoretical** |  | **Exp.** | **Theoretical** |  |
| **[Fe^3+^ + (Cat−2H^+^) + (Glu−2H^+^)]^−^** | ^12^C_11_H_11_NO_6_^54^Fe | 306.99 | 307.00 |  | 3.6 | 6.4 |  |
|  | ^12^C_11_H_11_NO_6_^56^Fe | 308.99 | 308.99 |  | 100 | 100 |  |
|  | ^12^C_11_H_11_NO_6_^57^Fe or ^12^C_10_^13^C_1_H_11_NO_6_^56^Fe | 309.99 | 310.00 |  | 9.8 | 14.2 |  |
|  | ^12^C_10_^13^C_1_H_11_NO_6_^57^Fe or ^12^C_9_^13^C_2_H_11_NO_6_^56^Fe | 310.99 | 311.00 |  | 1.6 | 1.2 |  |
